# P-810. Impact of Comorbid Heart Failure among Hospitalized Patients with *Escherichia coli* Sepsis: a United States Population-Based Cohort Study

**DOI:** 10.1093/ofid/ofae631.1002

**Published:** 2025-01-29

**Authors:** Carlo Gabriel C Casipit, Bruce Adrian C Casipit, Apoorva Subramanian, Yajur Arya, Arshi Syal, Mikaela Nikkola Jara-Tantoco, Phuuwadith Wattanachayakul

**Affiliations:** Jefferson-Einstein Hospital, Philadelphia, Pennsylvania; Jefferson-Einstein Hospital, Philadelphia, Pennsylvania; Jefferson-Einstein Hospital, Philadelphia, Pennsylvania; Jefferson-Einstein Hospital, Philadelphia, Pennsylvania; Jefferson-Einstein Hospital, Philadelphia, Pennsylvania; Jefferson-Einstein Hospital, Philadelphia, Pennsylvania; Jefferson-Einstein Hospital, Philadelphia, Pennsylvania

## Abstract

**Background:**

Sepsis-induced cardiomyopathy was deemed an independent predictor of increased risk for morbidity and mortality, especially for those individuals with pre-existing heart failure (HF). Although previous studies suggested that comorbid HF adversely impacted the outcomes of septic patients in general, there is paucity of data looking specifically at its impact among hospital patients with Escherichia coli (E. coli) sepsis.

**Figure:**
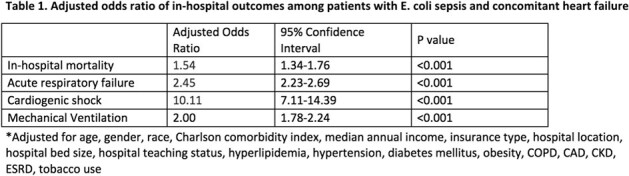

**Methods:**

We utilized the National Inpatient Sample (NIS) to identify patients with HF who were hospitalized for E. coli sepsis during the index hospitalization using appropriate ICD-10 codes between 2018-2020. We aim to investigate the impact of HF among E. coli septic patients based on in-hospital mortality, risk for acute respiratory failure and cardiogenic shock, and utilization of mechanical ventilation. A multivariable logistic regression analysis was used to calculate adjusted odds ratios (ORs) for the outcomes of interest.

**Results:**

A total of 93,896 hospitalized patients with E. coli sepsis were identified, of which 23.51% (22,075/93,896) had concomitant HF during the index hospitalization. The overall in-hospital mortality rate among patients with E. coli sepsis was 8.71% (8,178/93,896). Among those with concomitant HF, the mortality rate was significantly elevated to 13.05% (2,881/22,075, p= < 0.001). After adjusting for possible confounders, concomitant HF in patients with E.coli sepsis was found to be an independent predictor of increased risk for in-hospital mortality (aOR 1.54; 95% CI, 1.34-1.76; p=< 0.001), acute respiratory failure (aOR 2.45; 95% CI, 2.23-2.69; p=< 0.001), cardiogenic shock (aOR 10.11; 95% CI, 7.11-14.39; p=< 0.001), and utilization of mechanical ventilation (aOR 2.00; 95% CI, 1.78-2.24; p=< 0.001)

**Conclusion:**

Our analysis showed that HF among hospitalized E. coli sepsis patients was associated with an increased risk for adverse outcomes. This is likely due to the hemodynamic alterations in HF that are adversely impacted by sepsis treatment including fluid resuscitation, vasopressor use, and the advanced virulence factors of E. coli in adhesion, toxin production, and iron acquisition. Hence, identification of at-risk patients with HF is needed to mitigate its adverse impact among hospitalized patients with E. coli sepsis.

**Disclosures:**

**All Authors**: No reported disclosures

